# Personal

**Published:** 1903-08

**Authors:** 


### PERSONAL.

Dr. George Ben Johnston, the distinguished surgeon of Richmond, Va., was elected an honorary member of the Tazewell County (Va.) Medical Society, at its annual meeting, held June 16, 1903. Dr. Johnston is a native of Tazewell, and, no doubt, will appreciate and prize the distinction conferred upon him by the physicians of that famous county.

---

Dr. John Young Brown, Jr., of Saint Louis, Mo., has been appointed superintendent and surgeon-in-charge of the Saint Louis City Hospital. This is a most excellent appointment, Dr. Brown being an accomplished surgeon, as well as an administrative officer of ability and experience. He assumed his duties, May 13, 1903.

---

Dr. H. H. Glosser, University of Pennsylvania, 1899, who located in Buffalo last year, has removed from 1007 Elmwood Avenue, to 234 South Division Street. Telephone, Seneca 1727.

---

Dr. Hugo O. Pantzer, of Indianapolis, has been elected professor of gynecology in the Central College of Physicians and Surgeons, located in that city.

---

Major Albert H. Briggs, M. D., of Buffalo, surgeon 65th Regiment, N. Y. N. G., was elected a vice-president of the Association of Military Surgeons of the United States, at the twelfth annual meeting of that body, held at Boston, May 19, 20 and 21, 1903. This is a befitting recognition of the valuable services of Major Briggs, both to the national guard and the association.

---

Dr. James F. W. Ross, of Toronto, was elected president of the Ontario Medical Association, at its recent meeting, held at Toronto.

Dr. William H. Callahan, of Buffalo, has been appointed sanitary inspector of the Chautauqua Institute at Chautauqua, N. Y. He was graduated from the University of Buffalo in May last, and was inspector of tenements for the State Commission, in which capacity his services were much appreciated.

---

Dr. Charles Cary, of Buffalo, returned from an extended tour in Europe, during the latter days of July.

